# *Cucumis sativus* extract elicits chloride secretion by stimulation of the intestinal TMEM16A ion channel

**DOI:** 10.1080/13880209.2021.1949357

**Published:** 2021-08-06

**Authors:** Tultul Saha, Joydeep Aoun, Paramita Sarkar, Andrea J. Bourdelais, Daniel G. Baden, Normand Leblanc, John M. Hamlyn, Owen M. Woodward, Kazi Mirajul Hoque

**Affiliations:** a Pathophysiology Division, National Institute of Cholera & Enteric Diseases, Kolkata, India; b Department of Pharmacology, The Center for Cardiovascular Research, Center of Biomedical Research Excellence for Molecular and Cellular Signal Transduction in the Cardiovascular System, University of Nevada, Reno School of Medicine, Reno, NV, USA; c Center for Marine Science, University of North Carolina, Wilmington, NC, USA; d Department of Physiology, University of Maryland School of Medicine, Baltimore, MD, USA

**Keywords:** Loperamide-induced C57BL/6 constipation mouse model, T84 cells

## Abstract

**Context:**

Cucumber (*Cucumis sativus* Linn. [Cucurbitaceae]) is widely known for its purgative, antidiabetic, antioxidant, and anticancer therapeutic potential. However, its effect on gastrointestinal (GI) disease is unrecognised.

**Objective:**

This study investigated the effect of *C. sativus* fruit extract (CCE) on intestinal chloride secretion, motility, and motor function, and the role of TMEM16A chloride channels.

**Materials and methods:**

CCE extracts were obtained from commercially available cucumber. Active fractions were then purified by HPLC and analysed by high resolution mass spectrometry. The effect of CCE on intestinal chloride secretion was investigated in human colonic T84 cells, *ex vivo* mouse intestinal tissue using an Ussing chamber, and the two-electrode voltage-clamp technique to record calcium sensitive TMEM16A chloride currents in *Xenopus laevis* oocytes. *In vivo*, intestinal motility was investigated using the loperamide-induced C57BL/6 constipation mouse model. *Ex vivo* contractility of mouse colonic smooth muscles was assessed by isometric force measurements.

**Results:**

CCE increased the short-circuit current (ΔIsc 34.47 ± µA/cm^2^) and apical membrane chloride conductance (ΔI_Cl_ 95 ± 8.1 µA/cm^2^) in intestinal epithelial cells. The effect was dose-dependent, with an EC_50_ value of 0.06 µg/mL. CCE stimulated the endogenous TMEM16A-induced Cl^-^ current in *Xenopus laevis* oocytes. Moreover, CCE increased the contractility of smooth muscle in mouse colonic tissue and enhanced small bowel transit in CCE treated mice compared to loperamide controls. Mass spectrometry suggested a cucurbitacin-like analogue with a mass of 512.07 g/mol underlying the bioactivity of CCE.

**Conclusion:**

A cucurbitacin-like analog present in CCE activates TMEM16A channels, which may have therapeutic potential in cystic fibrosis and intestinal hypodynamic disorders.

## Introduction

Intestinal hydration primarily requires active Cl^-^ secretion through chloride channels present in the apical membrane (Frizzell et al. 1981; Begenisich and Melvin 1998). These include the cystic fibrosis transmembrane conductance regulator (CFTR) channel and the Ca^2+^ -activated Cl^-^ channel (CaCC). Classically, regulation of these ion channels occurs in response to agents that alter either cyclic nucleotides or intracellular calcium ([Ca^2+^]_i_), respectively (Hoque et al. 2010). Defective Cl^-^ transport by the intestinal epithelia has been described among patients with cystic fibrosis (CF). The pathogenesis of gastrointestinal (GI) disease in CF is thought to involve defective CFTR-mediated Cl^-^ secretion that results in a relatively dehydrated luminal environment. A major consequence of the altered luminal environment is the accumulation of mucus in the CF intestine that can lead to prolonged intestinal transit time, distal intestinal obstruction syndrome (DIOS) and chronic constipation (Sabharwal 2016). CF-associated constipation affects ∼47% of the CF population, and unfortunately it is not widely recognised. Furthermore, gastrointestinal motility disorders are frequently reported in CF (Olivier et al. 2015). Mutations in the CF gene likely lead to uniform loss of CFTR function although the severity of the disease differs among affected organs (Boat et al. 1989). These differences are, in part, due to compensatory expression of alternative, non-CFTR, Cl^-^ channels in the epithelium. Many studies have demonstrated that the gene expression of *ANO1* (TMEM16A) in oocytes and mammalian cells leads to the expression of a calcium-activated chloride channel (CaCC) and Cl^-^ currents (Caputo et al. 2008; Schroeder et al. 2008; Yang et al. 2008). It has been proposed that TMEM16A is the major CaCC expressed in the intestinal epithelium (Ousingsawat et al. 2009) and that this channel participates in intestinal Cl^-^ secretion (Flores et al. 2009). *ANO1* transcripts and TMEM16A are expressed robustly in gastrointestinal muscles, specifically in the interstitial cells of Cajal (ICC) in murine, non-human primate, and human GI tracts (Chen et al. 2007; Hwang et al. 2009; Gomez-Pinilla et al. 2009). Normal motor activity, such as peristalsis and segmentation in the GI tracts, is mediated via TMEM16A in the ICC of GI smooth muscles by the generation of electrical slow wave activity (Zhu et al. 2009). Thus, therapies targeting the activation of alternative Cl^-^ channels like TMEM16A, have received considerable attention for alleviating intestinal CF, as well as intestinal dynamic disorders such as constipation and constipation-predominant irritable bowel syndrome.

Small molecules from natural resources have shown potential for regulating CaCC mediated Cl^-^ secretion in intestinal epithelial cells (Zhu et al. 2020). Indeed, *Cucumis sativus* L. (Cucurbitaceae) has been used in Indian traditional medicine since ancient times. It is cultivated worldwide and a popular vegetable in tropical countries. Different parts of the plant *viz.* leaves, fruits and seeds produce several phytoconstituents with therapeutic benefits including several fatty acids, polyphenols, sterols and cucurbitacins. The latter are triterpenoid sterols that have a wide range of pharmacological actions including purgative, diuretic, anticancer, and anti-hyperglycemic activity (Mukherjee et al. 2013; Gill et al. 2010; Patil et al. 2012). In this study, we explored the bioactivity of CCE and identified a cucurbitacin-like analogue from HPLC-purified extracts. We studied the bioactivity of CCE in a number of model systems including the following: handling of intracellular Ca^2+^ in epithelial cells, activation of intestinal TMEM16A channels, active transepithelial Cl^-^ secretion, and GI transit time in a constipation mouse model and *ex vivo* on intestinal smooth muscle contraction. Taken together, our study demonstrates that a cucurbitacin-like analogue from *C. sativus* activates TMEM16A channels, stimulates intestinal Cl^-^ secretion, and augments muscle contractility and motor function. These actions imply possible utility for alleviating intestinal and intestinal hypodynamic disorders including that observed in CF.

## Materials and methods

### Reagents

Unless otherwise stated, all chemicals used in this study were obtained from Sigma-Aldrich. Cell culture media and foetal bovine serum (FBS) were purchased from Cell Clone and HiMedia, respectively. Penicillin-streptomycin was obtained from Invitrogen and CaCCinh-A01 was purchased from Calbiochem.

### Extract preparation and HPLC purification

Aqueous extracts were obtained by removing the skin of commercially available cucumbers (approx. weight 500 g cucumbers) from a local market and mashing the remaining matter. The crude extracts were filtered using a Buchner funnel with Whatman grade 1 filter and the filtrates were centrifuged at 10,000 *g* for 10 min at 4 °C. The supernatants were lyophilised and stored at −20 °C. The rate of extraction of crude CCE is 1.8–2.5 g/L of filtered aqueous extract. Further purification was performed by preparative reverse phase-high performance liquid chromatography (RP-HPLC) by dissolving it into acetonitrile-water (5:95 v/v). The extract was passed through a (2.2 cm × 25 cm; 5 µm) Vydac 218TP1022 reversed phase C18 column (Waters Corporation, Milford, MA) equilibrated with buffer A (0.1% trifluoroacetic acid, HPLC grade water, Merck) in a Breeze 2 HPLC system. A flow rate of 8 mL/min was maintained and the elute was monitored using a photo diode array detector. The separation was carried out using 100% buffer A for 8 min, followed by a linear gradient, 0–60% buffer B (0.05% trifluoroacetic acid, 100% acetonitrile, Merck), for 30 min; column temperature was 20 °C. The fraction containing the most prominent UV absorbing peaks was collected separately and concentrated by a Savant speed vac concentrator. The dried materials were dissolved in dimethyl sulfoxide (DMSO) to perform functional studies. For further investigation, a mass analysis was performed using a Xevo G2-XS QToF high resolution mass spectrometer (HRMS) in positive ion resolution mode controlled by MassLynx v4.1 software (Waters Corporation). Key parameters were the capillary voltage set to 2.5 kV, sampling cone set to 80 V, source offset set to 80 V, source temperature at 120 °C and desolvation gas flow set to 800 L/h.

### Cell culture

Human colonic T84 cells were routinely maintained in a ratio of Dulbecco’s modified Eagle’s medium (DMEM) and Ham’s F-12 medium supplemented with 10% foetal bovine serum (FBS), 100 units/mL penicillin, and 100 µg/mL streptomycin. T84 cells between passages 8 and 20 were seeded onto polycarbonate membrane, 12 mm Snapwell permeable support cell culture inserts (0.4 µm pore size; Costar), and grown for 10–15 days during which the media were changed every 48 h. Monolayer resistance was determined using an EVOM ohmmeter with STX2 electrodes (World Precision Instruments, Inc.) (Sheikh et al. 2013). IB3-1 cells were obtained from the American Type Culture Collection (Manassas, VA). The IB3-1 cell line is a compound heterozygote bronchial epithelial cell line from a CF patient containing one ΔF508 allele and one W1282× nonsense mutation allele. IB3-1 cells were cultured in LHC-8 medium (Invitrogen, Carlsbad, CA) supplemented with 5% FBS, 100 U/mL penicillin, 100 μg/mL streptomycin, 2 mM l-glutamine, and 1 μg/mL amphotericin B (Fungizone).

### Animal and tissue preparation

Three to four C57BL/6 male mice, 6–8 weeks of age, and fasted overnight, were used for each group in all intestinal Cl^-^ secretion, charcoal transit, and smooth muscle contraction experiments. Animal experiments and protocols were approved by the Institutional Animal Ethics Committee (NICED/CPCSEA/AW/225/IAEC-KMH/3). Following euthanasia by CO_2_ inhalation, the mouse colonic tissues were removed, and sero-musculature stripping was done on the top of an ice-cold glass plate. The mucosa was typically mounted on the snap well chambers (catalog number P2304; area 0.30 cm^2^; Physiologic Instruments Inc.) and transepithelial transport of ions was measured (Aoun et al. 2016).

### Transepithelial I_sc_ measurement

Monolayers were considered polarised and appropriately mounted in an Ussing chamber when resistance was equal to or greater than 1,500 Ω.cm^2^. The T84 cells grown on Snapwell inserts were mounted in an Ussing chamber for short circuit current (Isc) measurements, which were done at 37 °C with both sides of the monolayer immersed in an oxygenated HCO_3_-free solution containing (in mM) 140 NaCl, 5 KCl, 1 MgSO_4_, 2 CaCl_2_, 10 HEPES, and 10 glucose, pH 7.4. Fluid (5 mL) in each half of the chamber was connected via KCl agar bridges to voltage and current electrodes and clamped at 0 mV using a VCC MC6 multi-channel voltage-current clamp amplifier (Physiologic Instruments). Tetrodotoxin (TTX, 0.5 µM) was used to eliminate the possible neuronal influence on short circuit currents (Isc) and amiloride (10 µM) was used to inhibit epithelial sodium channels (ENaC) (Sheikh et al. 2013). The change in Isc induced by the treatment was expressed as the difference from the baseline to the steady state. The effects of CCE on apical membrane Cl^-^ conductance (I_Cl_) were assessed in T84 cell monolayers after permeabilization of the basolateral membrane with 50 µg/mL nystatin and the establishment of a basolateral to apical Cl^-^ concentration gradient according to our previously published protocol (Hoque et al. 2010).

### Two electrode voltage clamp (TEVC)

Two electrode voltage clamp recordings from *Xenopus laevis* oocytes were performed at room temperature in standard ND-96 Ringer’s solution (in mM: 96 NaCl, 2 KCl, 1 MgCl_2_, 1.8 CaCl_2_, 5 HEPES, pH 7.5) (Woodward et al. 2010). To increase the conductance of endogenous TMEM16A currents, oocytes were treated with HPLC-purified CCE in the ND-96 bath solution. A holding potential of −40 mV was used on all oocytes.

### Intracellular calcium measurement

IB3-1 cells were seeded on a collagen-coated cover glass. Cells were rinsed with non-supplemented media, then loaded with Fura-2/AM (Invitrogen, USA) and bathed in a Ca^2+^-free solution (in mM: 120 NaCl, 4.5 KCl, 1 EGTA, 2 MgCl_2_, 10 HEPES, pH 7.4). Images were acquired on a Zeiss microscope (Zeiss Observer A1, Germany) and a FluorArc was used to excite the cells at 340 and 380 nm, controlled via a Sutter (USA) Lamda 10-2 controller and filter wheel assembly, and the emission measured at 510 nm. All images were acquired using a Coolsnap CF CCD camera (Photometrics, USA) and fluorescence at each wavelength measured once every 5 sec. Image acquisition, analysis, and wheel control were performed by IPLab Software (BD Biosciences, USA). Cells were treated with 0.12 µg/mL of HPLC fraction of CCE in ringer’s solution with 3–5 mM CaCl_2_. Each data point was normalised to the individual initial fluorescence value, ΔF/F= (F340/F380)/(F340/F380_initial_) to bring all the response curves to the same pre-treatment starting point (Woodward et al. 2010).

### Intestinal motility measurements

Constipation was induced in C57BL/6 mice through oral administration of 3 mg/kg loperamide hydrochloride daily for 5 to 6 days. One group of loperamide-treated mice was administered with HPLC-purified CCE (5 mg/kg, orally). The control animals were administered only phosphate buffer solutions (PBS). Animals were fasted for 24 h before the experiment and divided into three groups administered respectively with PBS, loperamide, and loperamide plus CCE. After 15 min of CCE administration, the animals were fed 0.3 mL charcoal meal (consisting of 10% vegetable charcoal in 5% gum acacia). After 30 min of charcoal administration, the animals were sacrificed by cervical dislocation and the total intestines were isolated. The charcoal transit ratio was calculated as the percentage of distance transited by the charcoal relative to the total length of the small intestine (pyloric sphincter to the cecum).

### Muscle contraction

Muscle contraction was measured by an isometric force transducer (Grass Technologies, West Warwick, RI). The colon segment (∼2 cm) of adult mice was carefully removed and tied with a silk thread to each end to hang vertically into an organ bath containing Krebs solution containing (in mM): 120 NaCl, 4.2 KCl, 1.2 MgSO_4_, 0.6 KH_2_PO_4_, 25 NaHCO_3_, 11.1 glucose, and 1.8 CaCl_2_ (pH 7.4). The organ bath was aerated with 95% O_2_, 5% CO_2_; temperature was maintained at 37 °C for the duration of the experiment. After a stable contraction was obtained, experiments were performed in the presence of carbachol (CCH) and purified CCE fraction. The effect of CaCCinhA01 (TMEM16A inhibitor) on tension evoked by CCE was also evaluated. All contractions were recorded using AcqKnowledge data acquisition software version 3.9.1 (Biopac Systems, Goleta, CA) (Forrest et al. 2012).

### Statistical analysis

Statistical data and graphic analysis were performed using Origin 6.0 (OriginLab, Northampton, MA, USA) software. Data are expressed as mean ± standard error (SE). Statistical significance was determined by using paired or unpaired *t*-test, multiple *t*-test corrected with the Holm-Sidak method, and ANOVA as applicable. A *p* value <.05 was considered statistically significant as indicated.

## Results

### Activation of Cl^-^ secretion by *C. sativus* extract (CCE) in human colonic T84 cells

**Figure 1.**
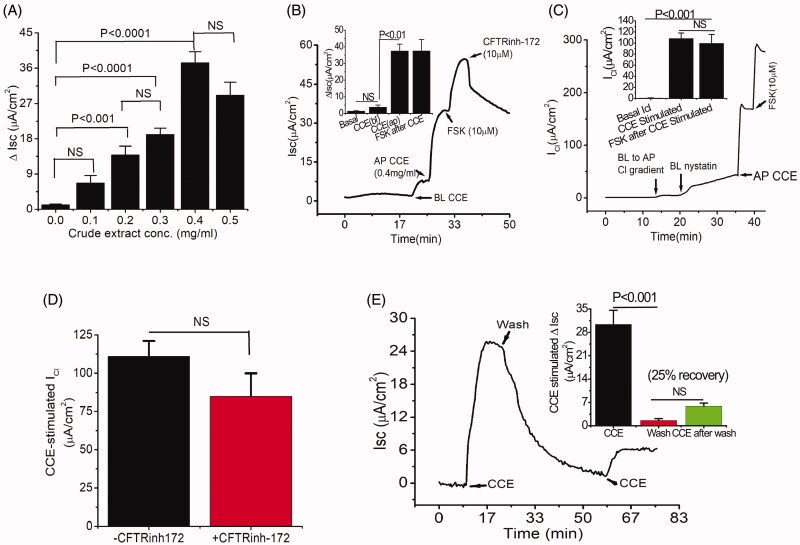
Bioactive constituent from crude cucumber extract (CCE) stimulates apical Cl^-^ conductance without altering CFTR conductance. (A) Summarised data showing Isc stimulation by crude aqueous extract at different concentrations (mg/mL). Values are means ± S.E. *n* = 3–5. One-way ANOVA with Holm-Sidak’s multiple-comparisons test was used to compare mean values of different dose of CCE effect against baseline and between doses as indicated. (B) Apical (AP), but not basolateral (BL) additions of CCE (0.4 mg/mL) induced increases in Isc. T84 cells grown on filters were mounted in Ussing chambers and exposed to CCE as indicated and then forskolin (FSK) was added after a steady state had been reached. CFTR-inh172 inhibits forskolin but not CCE stimulated Isc. Inset: Summary data showing average ΔIsc recorded under various conditions (*n* = 5). (C) CCE responses in nystatin-permeabilized monolayers. T84 monolayers were treated with basolateral nystatin and in the presence of an apical to basolateral chloride gradient under voltage clamp condition; CCE induced an apical Cl^-^ current I_Cl_. The inset shows the average current induced by CCE (*n* = 3). (D) Summary data depicting average CCE stimulated ΔI_Cl_ in presence and absence of CFTRinh-172. (E) Representative record showing recovery of the response of a T84 cell monolayer exposed to CCE after washing out the extract. Inset, summarised data showing the recovery of Isc response after second application of CCE after wash. Statistical significance in B, C, D, and E determined with paired t-test. NS: not statistically significant.

In CF, TMEM16A-mediated secretion is intact and provides a therapeutic target to circumvent Cl^-^ secretion defects. Thus, the discovery of novel compounds that would stimulate TMEM16A without adverse effects could compensate for the loss of Cl^-^ secretion and improve the adverse phenotype associated with CF. We performed bioactivity analysis of crude CCE by measuring transepithelial Isc in T84 monolayers in Ussing chamber. Crude CCE produced a dose-dependent increase in Isc when applied on the apical side with a maximal effect seen at a concentration of 0.4 mg/mL (Figure 1(A)). This concentration of the crude CCE was chosen in the succeeding experiments to monitor the impact of various interventions. In contrast, basolateral addition of CCE induced a negligible increase in Isc. After addition of apical CCE, cells were further treated with the adenylate cyclase activator forskolin (FSK, 10 µM) to activate CFTR mediated Cl^-^ secretion. A steady state response (cAMP mediated Cl^-^ secretion) was observed, that was completely inhibited by the CFTR inhibitor CFTRinh-172 (10 µM). However, the inhibitory effect of CFTRinh-172 was insensitive to CCE-stimulated Isc (Figure 1(B)). We next examined whether the increase of Isc by CCE was involved in the activation of apical membrane Cl^-^ channels by measuring the apical membrane Cl^-^ conductance (I_Cl_). For these experiments, basolateral membranes were depolarised with high K^+^ solution in the presence of a basolateral to apical Cl^-^ gradient. As shown in Figure 1(C), administration of crude CCE (0.4 mg/mL) to the apical side of basolateral permeabilized T84 cells activated a significant I_Cl_; yet in response to FSK, the cAMP stimulated I_Cl_ was not altered. This observation was further evaluated by pre-treating the cells with CFTRinh-172. We found that the effect of CCE was not altered significantly in the presence of CFTRinh-172 (Figure 1(D)), suggesting that CCE targets another Cl^-^ conductance. To further determine whether the increase in Isc was reversible, CCE was washed out from the apical chamber and Isc gradually returned to near baseline level; a second application of CCE still triggered a current (Figure 1(E)), albeit less pronounced (25% recovery), suggesting that the effect was reversible and repeatable. These observations strongly support the assertion that crude CCE activated a CFTR-independent chloride secretion via an interaction with a “receptor” present on the luminal membrane.

### Bioactivity-directed fractionation of CCE by HPLC

**Figure 2.**
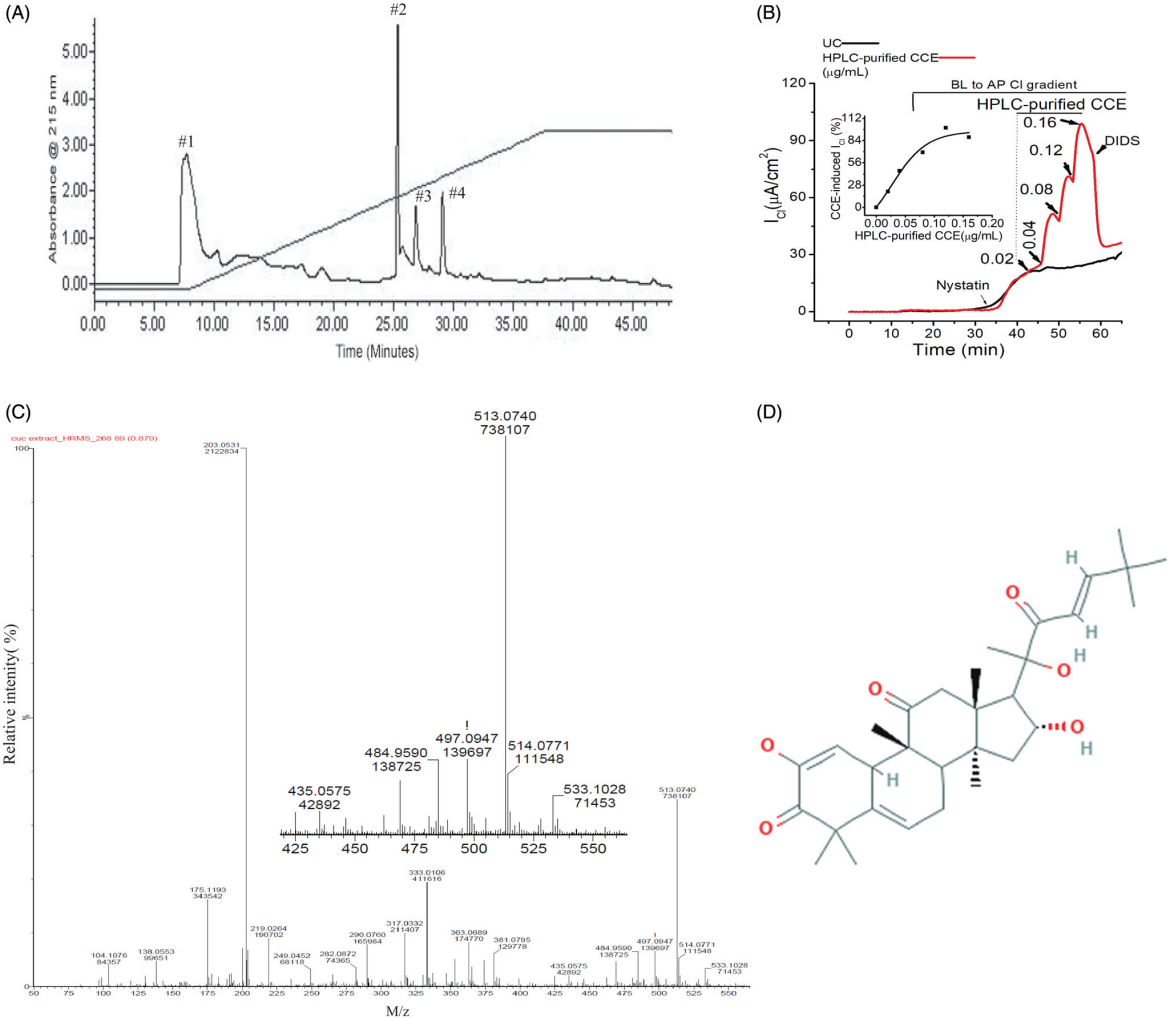
Separation of an active fraction of the CCE and its effect on apical Cl^-^ conductance in T84 cells. (A) Preparative HPLC chromatogram showing fractions from CCE using water-acetonitrile gradient elution. (B) Response to cumulative addition of purified CCE fraction on nystatin-permeabilized T84 monolayers in the presence of a basolateral to apical chloride gradient; the CCE-induced Cl^-^ current was inhibited by an apical application of the non-specific CaCC blocker DIDS (100 μM). This record is representative of two independent experiments; UC stands for untreated control. Inset, kinetics of HPLC-purified CCE stimulated I_Cl_ in T84 cells showing EC_50_ is 0.06 µg/ml. (C) Time-of-flight high resolution mass spectral scan of the purified fraction (HPLC fraction #2), revealed a CCB-like entity with a prominent protonated molecular ion at m/z 513.0740. Inset shows the expanded region of interest. (D) Suggested 2 D structure for the bioactive CCB-like compound corresponding to the elemental formula C_31_H_44_O_6_ (PubChem CID 58599137).

Crude CCE was fractionated further by RP-HPLC using a water-acetonitrile gradient to obtain discrete compounds present in the aqueous extract. Four prominent ultraviolet absorbing fractions were obtained and collected (Figure 2(A)). Functional screening of these four fractions revealed that fraction #2 with a retention time of 26 min, evoked an increase in transepithelial Cl^-^ current in nystatin-permeabilized T84 monolayers (Figure 2(B)). The stimulatory effect of this fraction (HPLC-purified CCE) was dose-dependent, with a threshold concentration of 0.04 µg/mL and a maximal effect observed at 0.12 µg/mL (Figure 2(B)). These responses were completely inhibited by DIDS (4,4′-diisothiocyanato-stilbene-2,2′-disulfonic acid), a broad spectrum Ca^2+^ activated Cl^-^ channel blocker that is known to inhibit TMEM16A. A higher concentration of the fraction (0.16 µg/mL) failed to activate the Cl^-^ current further. Analysis shows that the EC_50_ of this effect was 0.06 µg/mL (Figure 2(B) inset). The relative activity of the HPLC-purified CCE (0.12 µg/mL) was ∼3000-fold greater than that produced by the crude (0.4 mg/mL) fraction. The LC-MS spectra of the HPLC-purified CCE showed 7 prominent protonated molecular ions ranging from 175–533 m/z. Among these ions the most abundant species were seen at 203 and 513 m/z with the latter having an exact monoisotopic mass (M + H^+^) of 513.0740 m/z (Figure 2(C)). We suggest that one of the 7 prominent ions was likely responsible for the bioactivity in intestinal epithelia. Based on the abundance of molecular ions in scans of the bioactive fraction #2, the closest match for the 513 m/z ion in PubChem was for Compound CID 58599137 i.e., (9 *R*,13*R*,14*S*,16*R*)-2,16-dihydroxy-17-[(*E*,2*R*)-2-hydroxy-6,6-dimethyl-3-oxohept-4-en-2-yl]-4,4,9,13,14-pentamethyl-8,10,12,15,16,17-hexahydro-7H cyclopenta[a]phenanthrene-3,11-dione having an elemental formula of C_31_H_44_O_6_ (Figure 2(D)).

### Activation of TMEM16A by bio-active molecules of CCE

**Figure 3.**
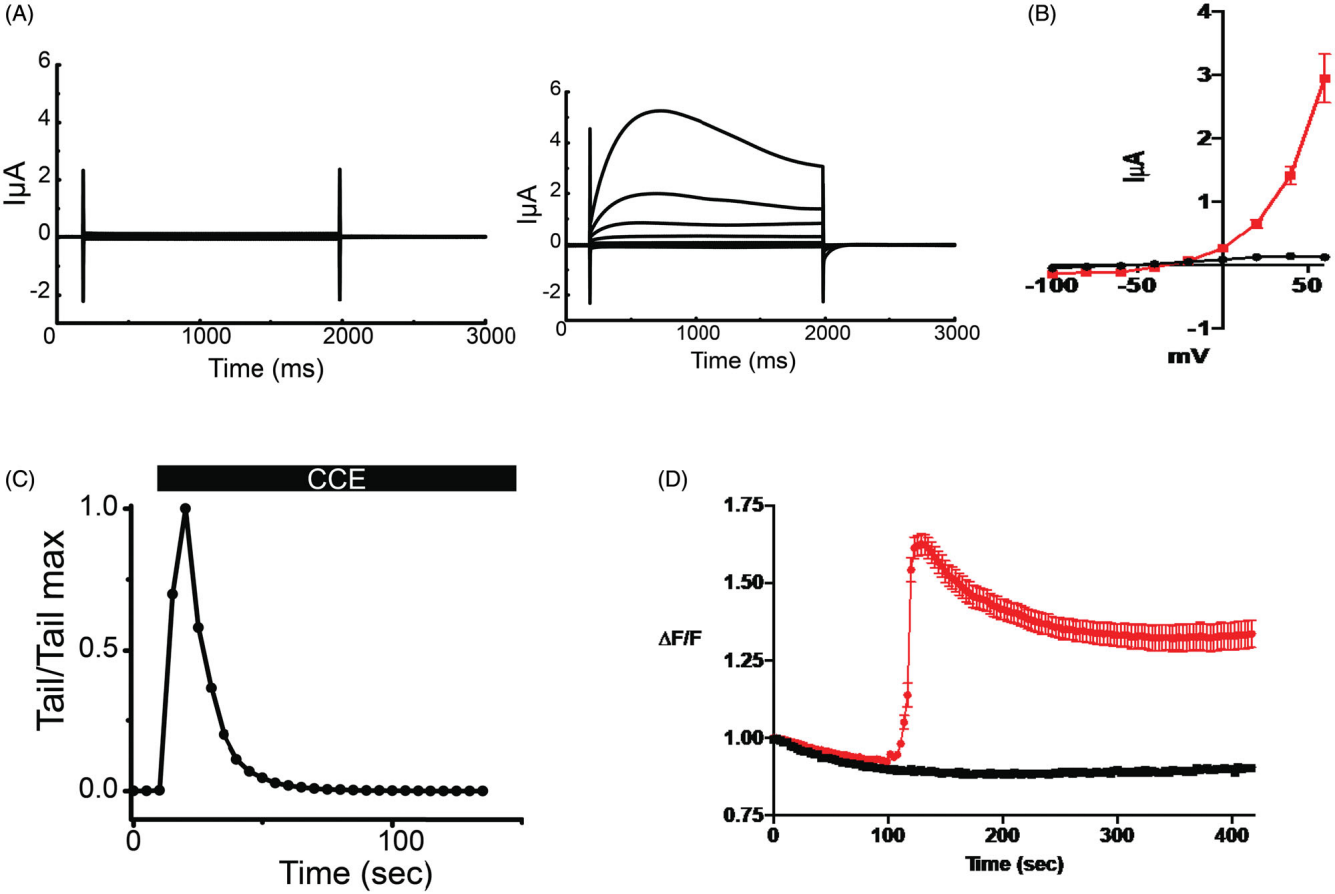
CCE activates TMEM16A mediated current in Xenopus laevis oocytes and releases intracellular calcium in human epithelial cells. (A) Whole cell currents in Xenopus oocytes held at −40 mV and subjected to +20 mV voltage steps before (left) and after (right) the addition of the bioactive HPLC fraction of CCE (0.12 μg/mL). (B) Current voltage relation of whole cell currents in oocytes. Current density at the end of the traces (A) were averaged and plotted against membrane potential. Data (± SEM, *n* = 5, evaluated with t-test, *p < .0001). CCE (red plot) activates current with outward rectification and amplitudes consistent with the well characterized TMEM16A channel endogenous to Xenopus oocytes. (C) The activation of the TMEM16A currents by the CCE is transitory, as evident by declining outward current amplitudes at +60 mV over time and the instantaneous tail currents at – 40mV. (D) Measuring relative changes in intracellular Ca^2+^ in human bronchial epithelial cells (IB3-1) with CCE and without (*n* = 3 cover slips and 32 cells for each) in zero Ca^2+^ bath using Fura-2. ΔF/F is the measured F340/F380 ratio normalized to the initial F measurement (see methods). CCE significantly increased intracellular Ca^2+^ as determined by Two-way ANOVA, *p*<0.0001).

To examine the effect of bio-active molecule from HPLC fractions on TMEM16A, we took advantage of the robust endogenous expression of this channel in *Xenopus laevis* oocytes. Using the two-electrode voltage clamp technique, oocytes were held at −40 mV and then subjected to +20 mV voltage clamp steps ranging from −100 to +60 mV. Only small voltage- and time-independent leak currents were recorded in the absence of the CCE (Figure 3(A), left traces]. The purified CCE (0.12 µg/mL) activated a robust membrane current exhibiting outward rectification and amplitudes consistent with the well-characterized endogenous TMEM16A-induced Cl^-^ current in *Xenopus laevis* oocytes (Figure 3(A), right traces and Figure 3(B)). The activation of the I_TMEM16A_ by the purified CCE was transient, as evidenced by the time-dependent decline of the outward current at +60 mV and the instantaneous tail current upon return to −40 mV (Figure 3(C)). To discern the involvement of intracellular Ca^2+^ on CCE-mediated Cl^-^ channel activation, we measured intracellular Ca^2+^ levels in the IB-3 cell line derived from a cystic fibrosis patient by monitoring fluorescence intensity using the ratiometric dye Fura-2 in the presence of the purified fraction. As shown in Figure 3(D), the cells were preincubated in Ca^2+^-free medium and the purified fraction of CCE caused a rapid and transient rise of intracellular Ca^2+^ that stabilised to a plateau level that was significantly higher than baseline. These results suggest that CCE activates TMEM16A, most likely by releasing Ca^2+^ from internal stores, and that this mechanism probably underlies the ability of TMEM16A to enhance Cl^-^ secretion.

### CCE induces intestinal contractility and motor function

**Figure 4.**
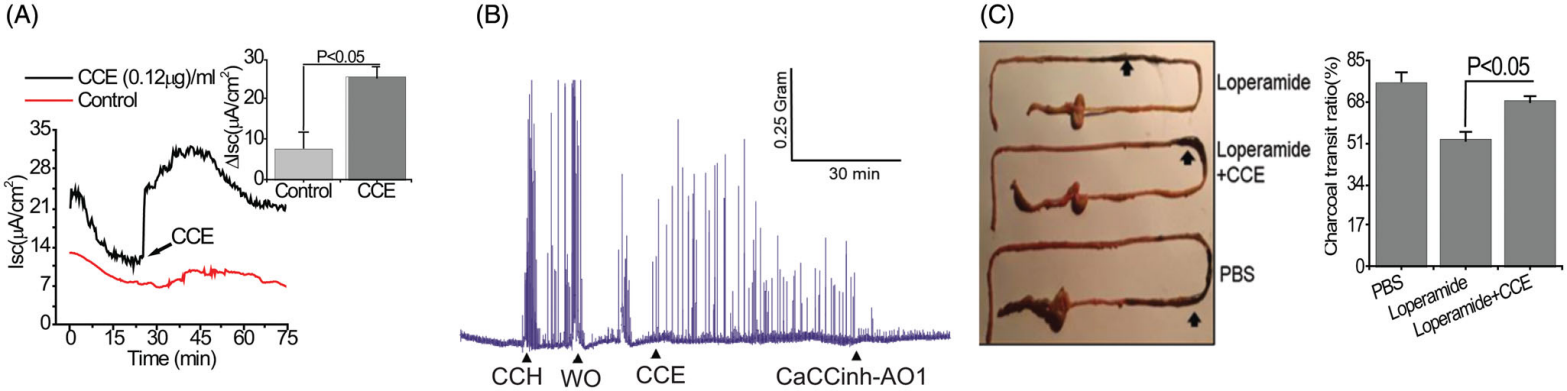
Stimulation of Cl^-^ secretion and smooth muscle contraction by CCE in mouse intestinal tissue. (A) Representative data showing the effect of partially purified CCE on Isc change in wild type mouse (C57BL/6) colonic tissue (*n* = 4). The inset shows summary data from four independent experiments. Data are means ± S.E. (B) Representative traces showing the effect of CCE on smooth muscle contraction in isolated mouse colon and its inhibition by CaCCinh-A01 (*n* = 3). (C) Representative image of isolated mouse intestinal tracts showing movement of activated charcoal after oral gavage of PBS (positive control), Loperamide in the presence or absence of CCE. Bar graph showing a summary of the % charcoal transit ratio under the three conditions tested. Data are the means ± S.E of three independent experiments.

Because TMEM16A channels can promote pacemaker activity that augments smooth muscle contraction in the gastrointestinal tract (Hwang et al. 2009), experiments were performed *in vivo* to explicate the stimulatory effect of the bio-active material on the spontaneous contractility of colonic smooth muscles. Similar to its effects on cultured GI epithelial cells, Figure 4(A) shows that the purified fraction of CCE was also able to trigger Isc in mouse colonic tissue. CCH (50 µM) increased the frequency of spontaneous phasic contractions in intact mouse colonic strips, an effect that disappeared after washout. Application of the purified fraction of CCE produced a similar effect to CCH on contractions, which was abolished by exposure to the TMEM16A inhibitor CaCCinh-A01 (10 µM) (Figure 4(B)). The motor function of upper GI tracts was estimated by small bowel transit *in vivo* by determining the charcoal transit. For these experiments, the loperamide-treated constipation mouse model was used because loperamide is known to inhibit colonic smooth muscle contractions, delay intestinal luminal transit, and promote constipation (Kojima et al. 2009). As shown in Figure 4(C), CCE caused a significant enhancement of the travelled distances of luminal charcoal in comparison to control mice only treated with loperamide (loperamide control: 44 ± 6.24 *vs*. CCE: 67.33 ± 4.63, *p* < .05); in fact, the travelled distance of luminal charcoal was similar to that measured in mice unexposed to loperamide. Together, these findings indicate that purified CCE may offer a beneficial role in augmenting spontaneous motility and contractility by activating TMEM16A channels.

## Discussion

Plants produce diverse compounds and are an important source for promising drug candidates with highly selective biological activities. The primary purpose of this study was to attempt to rescue intestinal Cl^-^ secretion, GI motility and motor function via the activation of TMEM16A channels by a natural compound that may be useful for the treatment of CF-related intestinal phenotypes.

In this study, we detected bioactivity in crude as well as HPLC fractions derived from an aqueous fruit extract of *C. sativus*. The bioactive material is a potent intracellular Ca^2+^ mobilising agonist that activated TMEM16A channels. This is supported by our finding that the purified HPLC fraction activated the characteristic outwardly rectifying TMEM16A Cl^-^ current in *Xenopus laevis* oocytes (Callamaras and Parker 2000). The active fraction stimulated an increase of [Ca^2+^]_i_ in IB3 cells, a compound heterozygote human epithelial CF cell line. Critically, in colonic T84 cells, the bioactive fraction had no effect on the Isc when applied from the basolateral side at any concentration whereas it produced a robust sustained Cl^-^ conductance upon apical administration. This supports the assertion that CCE interacts specifically with “receptors” that are located predominantly at the apical membrane. The HPLC purified fraction evoked a dose-dependent Cl^-^ conductance in basolateral permeabilized T84 cells, which was blocked by the administration of the TMEM16A inhibitor DIDS (Figure 2(B)). Although our results do not allow us to determine the molecular mechanism of this effect and the identity of the “receptors”, they do support the suggestion that the probable mechanism is not direct activation of TMEM16A, but instead involves pathways that raise [Ca^2+^]_i_ as a result of a G-protein coupled receptor (GPCR) activation on the luminal membrane.

Members of the Cucurbitacea family are noted for their beneficial laxative effect and also the potential for toxicity mediated by their cucurbitacin (CCB) content (Gry et al. 2006). Their beneficial laxative action is ascribed typically in the lay literature to their high-water content but may be mediated also by other factors that stimulate GI contractility including CCBs. Over 19 CCBs and numerous structurally CCB-like entities have been described and some (CCB-A, B, D, E, I) as well as their analogues (e.g., hemslecin A) are available in pure form commercially. Accordingly, we expected to find one or more of the known CCBs in the bioactive fraction we obtained from CCE. Most known CCBs and CCB-related entities have in common the classic four ring member steroid nucleus with various additions to the A and/or the D rings that lead to variations in molecular weights ranging from 344.4 to 626.6 g/mole. In addition, they have a complex stereochemistry that is not easily elucidated by mass spectrometry. Of relevance to the present work, there are numerous known CCBs in the mass range of 514 to 518 g/mol and we were surprised that, among these and related CCBs, none have molecular weights that match any of the more interesting prominent molecular ions (*m* + H^+^/*z* = 435, 484, 497, 513, 514, 533) we observed in the bioactive fraction (Figure 2(C)). On the basis of mass, the closet known curcubitacin is CCB-I with an average mass of 514.6 g/mol. Our finding of an abundant protonated monoisotopic molecular ion at 513.0740 (Figure 2(C) and inset) in the bioactive fraction suggested previously identified CCB-like compound named (9 *R*,13*R*,14*S*,16*R*)-2,16-dihydroxy-17-[(*E*,2*R*)-2-hydroxy-6,6-dimethyl-3-oxohept-4-en-2-yl]-4,4,9,13,14-pentamethyl-8,10,12,15,16,17-hexahydro-7H-cyclopenta[a]phenanthrene-3,11-dione) having an elemental formula of C_31_H_44_O_6_ and a computed average mass of 512.69 g/mol. However, the calculated exact monoisotopic mass of this compound is 512.313789 (i.e., M + H^+^ = 513.321065 *m/z*), and the observed high-resolution mass for the protonated molecular ion in Figure 2(C) was similar but not identical at 513.0740 *m/z*. Nevertheless, based upon the preliminary analytical data available and the results from other reported bio-active constituents of CCE extracts, it plausible that the bioactive material we detected is a novel CCB-like analogue; it differs only minimally in mass from other known bio-active CCB molecules and other curcubitacins such as CCB-B that stimulate [Ca^2+^]_i_ via activation of GPCR/IP_3_-receptor (Kim et al. 2018). In this study, the purified fraction also evoked a rise in [Ca^2+^]_i_ in IB3-1 epithelial cells derived from a CF patient. However, our findings, while functionally implicating a novel CCB-like entity as the basis for the bioactivity we observed. Neither prove nor exclude this possibility at this stage of investigation and further work will be required to identify the bioactive material conclusively.

Patients with CF often suffer from gastrointestinal dysmotility and prolonged transit time. The TMEM16A channel was identified as a new, selective molecular marker for all interstitial cells of Cajal (ICCs) in the intestine. These cells coordinate phasic contractions of smooth muscle cells such as peristalsis and segmentation in the gastrointestinal tract, with normal motor activities, by generating electrical slow waves (Gomez-Pinilla et al. 2009). This raises the important question as to whether the CCE-induced rise in [Ca^2+^]_i_ could translate into TMEM16A stimulation in the ICC that would be beneficial in the treatment of CF patients such as constipation and dysmotility. We observed that CCE accelerated intestinal transit by 53% in the loperamide-treated constipation mouse model compared to loperamide alone (Figure 4(C)). Our results further showed that the bio-active material activated intestinal smooth muscle contraction in isolated mouse colon as shown in Figure 4(B). Together, these data support the role of TMEM16A channels in the ICC and its activation by our novel bioactive material. Further, our results imply that the bioactive compound may be of therapeutic benefit in CF by stimulating TMEM16A channels, enhancing transepithelial ion flux, water movement, and GI motility that collectively should improve bowel transit time.

## Conclusions

This study detected a novel bio-active compound from fruit extract of *C. sativus* that increases intracellular Ca^2+^ and activates TMEM16A mediated Cl^-^ secretion and thereby may rescue defective intestinal Cl^-^ secretion and GI motility. We suggest that CCB-like entities may be useful lead compounds for the development of novel, orally active, therapeutic agents for CF-related as well as unrelated intestinal disorders.

## References

[CIT0001] Aoun J, Hayashi M, Sheikh IA, Sarkar P, Saha T, Ghosh P, Bhowmick R, Ghosh D, Chatterjee T, Chakrabarti P, et al. 2016. Anoctamin 6 contributes to Cl^-^ secretion in accessory cholera enterotoxin (Ace)-stimulated diarrhea: an essential role for phosphatidylinositol 4,5-bisphosphate (PIP_2_) signaling in cholera. J Biol Chem. 291(52):26816–26836. 27799301 10.1074/jbc.M116.719823 PMC5207189

[CIT0002] Begenisich T, Melvin JE. 1998. Regulation of chloride channels in secretory Epithelia. J Membr Biol. 163(2):77–85. 9592072 10.1007/s002329900372

[CIT0003] Boat TF, Welsh MJ, Beaudet AL. 1989. The metabolic and molecular bases of inherited disease. In: Cystic fibrosis in the metabolic basis of inherited disease. 6th ed. New York: McGraw-Hill; p. 2649–2680.

[CIT0004] Callamaras N, Parker I. 2000. Ca^2+^-dependent activation of Cl^-^ currents in Xenopus oocytes is modulated by voltage. Am J Physiol Cell Physiol. 278(4):C667–C675. 10751316 10.1152/ajpcell.2000.278.4.C667

[CIT0005] Caputo A, Caci E, Ferrera L, Pedemonte N, Barsanti C, Sondo E, Pfeffer U, Ravazzolo R, Moran OZ, Galietta LJV. 2008. TMEM16A, a membrane protein associated with calcium-dependent chloride channel activity. Science. 322(5901):590–594. 18772398 10.1126/science.1163518

[CIT0006] Chen H, Ordög T, Chen J, Young DL, Bardsley MR, Redelman D, Ward SM, Sanders KM. 2007. Differential gene expression in functional classes of interstitial cells of Cajal in murine small intestine. Physiol Genomics. 31(3):492–509. 17895395 10.1152/physiolgenomics.00113.2007

[CIT0007] Flores CA, Cid LP, Sepúlveda FV, Niemeyer MI. 2009. TMEM16 proteins: the long-awaited calcium-activated chloride channels? Braz J Med Biol Res. 42(11):993–1001. 19784506 10.1590/s0100-879x2009005000028

[CIT0008] Forrest AS, Joyce TC, Huebner ML, Ayon RJ, Wiwchar M, Joyce J, Freitas N, Davis AJ, Ye L, Duan DD, et al. 2012. Increased TMEM16A-encoded calcium-activated chloride channel activity is associated with pulmonary hypertension. Am J Physiol Cell Physiol. 303(12):C1229–C1243. 23034390 10.1152/ajpcell.00044.2012 PMC3532492

[CIT0009] Frizzell RA, Welsh MJ, Smith PL. 1981. Electrophysiology of chloride-secreting epithelia. Soc Gen Physiol Ser. 36:137–145. 6116284

[CIT0010] Gill NS, Bajwa J, Sharma P, Dhiman K, Sood S, Sharma PD, Singh B, Bali M. 2010. Evaluation of antioxidant and antiulcer activity of traditionally consumed *Cucumis melo* seeds. J Pharmacol Toxicol. 6(1):82–89.

[CIT0011] Gomez-Pinilla PJ, Gibbons SJ, Bardsley MR, Lorincz A, Pozo MJ, Pasricha PJ, Rijn MVD, West RB, Sarr MG, Kendrick ML, et al. 2009. Ano1 is a selective marker of interstitial cells of Cajal in the human and mouse gastrointestinal tract. Am J Physiol Gastrointest Liver Physiol. 296(6):G1370–G1381. 19372102 10.1152/ajpgi.00074.2009 PMC2697941

[CIT0012] Gry J, Saborg I, Christer Anderson H. 2006. Cucurbitacins in plant food. TemaNord: 556. Copenhagen: Nordic Council of Ministers. ISBN 92-893-1381-1.

[CIT0013] Hoque KM, Woodward OM, van Rossum DB, Zachos NC, Chen L, Leung GPH, Guggino WB, Guggino SE, Tse CM. 2010. Epac1 mediates protein kinase A-independent mechanism of forskolin-activated intestinal chloride secretion. J Gen Physiol. 135(1):43–58. 20038525 10.1085/jgp.200910339 PMC2806414

[CIT0014] Hwang SJ, Blair PJA, Britton FC, O'Driscoll KE, Hennig G, Bayguinov YR, Rock JR, Harfe BD, Sanders KM, Ward SM. 2009. Expression of anoctamin 1/TMEM16A by interstitial cells of Cajal is fundamental for slow wave activity in gastrointestinal muscles. J Physiol. 587(Pt 20):4887–4904. 19687122 10.1113/jphysiol.2009.176198 PMC2770154

[CIT0015] Kim KH, Lee IS, Park JY, Kim Y, An EJ, Jang HJ. 2018. Cucurbitacin B induces hypoglycemic effect in diabetic mice by regulation of AMP-activated protein kinase alpha and glucagon-like peptide-1 via bitter taste receptor signaling. Front Pharmacol. 9:1071. 30298009 10.3389/fphar.2018.01071 PMC6161541

[CIT0016] Kojima R, Doihara H, Nozawa K, Kawabata-Shoda E, Yokoyama T, Ito H. 2009. Characterization of two models of drug-induced constipation in mice and evaluation of mustard oil in these models. Pharmacology. 84(4):227–233. 19752586 10.1159/000236524

[CIT0017] Mukherjee PK, Nema NK, Maity N, Sarkar BK. 2013. Phytochemical and therapeutic potential of cucumber. Fitoterapia. 84:227–236. 23098877 10.1016/j.fitote.2012.10.003

[CIT0018] Olivier AK, Gibson-Corley KN, Meyerholz DK. 2015. Animal models of gastrointestinal and liver diseases. Animal models of cystic fibrosis: gastrointestinal, pancreatic, and hepatobiliary disease and pathophysiology. Am J Physiol Gastrointest Liver Physiol. 308(6):G459–G471. 25591863 10.1152/ajpgi.00146.2014 PMC4360044

[CIT0019] Ousingsawat J, Martins JR, Schreiber R, Rock JR, Harfe BD, Kunzelmann K. 2009. Loss of TMEM16A causes a defect in epithelial Ca^2+^-dependent chloride transport. J Biol Chem. 284(42):28698–28703. 19679661 10.1074/jbc.M109.012120 PMC2781414

[CIT0020] Patil MVK, Kandhare AD, Bhise SD. 2012. Effect of aqueous extract of *Cucumis sativus* Linn. fruit in ulcerative colitis in laboratory animals. Asian Pac J Trop Biomed. 2(2):S962–S969.

[CIT0021] Sabharwal S. 2016. Gastrointestinal manifestations of cystic fibrosis. Gastroenterol Hepatol (NY). 12:43–47. PMC4865785 27330503

[CIT0022] Schroeder BC, Cheng T, Jan YN, Jan LY. 2008. Expression cloning of TMEM16A as a calcium-activated chloride channel subunit. Cell. 134(6):1019–1029. 18805094 10.1016/j.cell.2008.09.003 PMC2651354

[CIT0023] Sheikh IA, Koley H, Chakrabarti MK, Hoque KM. 2013. The Epac1 signaling pathway regulates Cl^-^ secretion via modulation of apical KCNN4c channels in diarrhea. J Biol Chem. 288(28):20404–20415. 23720748 10.1074/jbc.M113.467860 PMC3711306

[CIT0024] Woodward OM, Li Y, Yu S, Greenwell P, Wodarczyk C, Boletta A, Guggino WB, Qian F. 2010. Identification of a polycystin-1 cleavage product, P100, that regulates store operated Ca^2+^ entry through interactions with STIM1. PLoS One. 5(8):e12305. 20808796 10.1371/journal.pone.0012305 PMC2925899

[CIT0025] Yang YD, Cho H, Koo JY, Tak MH, Cho Y, Shim WS, Park SP, Lee J, Lee B, Kim BM, Raouf R, et al. 2008. TMEM16A confers receptor-activated calcium-dependent chloride conductance. Nature. 455(7217):1210–1215. 18724360 10.1038/nature07313

[CIT0026] Zhu MH, Kim TW, Ro S, Yan W, Ward SM, Koh SD, Sanders KM. 2009. A Ca^2+^-activated Cl^-^ conductance in interstitial cells of Cajal linked to slow wave currents and pacemaker activity. J Physiol. 587(Pt 20):4905–4918. 19703958 10.1113/jphysiol.2009.176206 PMC2770155

[CIT0027] Zhu X, Zhang W, Jin L, Zhang G, Yang H, Yu B. 2020. Inhibitory activities of curzerenone, curdione, furanodienone, curcumol and germacrone on Ca^2+^-activated chloride channels. Fitoterapia. 147:104736. 33010370 10.1016/j.fitote.2020.104736

